# Extracellular Vesicle-Mediated Secretion of HLA-E by Trophoblasts Maintains Pregnancy by Regulating the Metabolism of Decidual NK Cells

**DOI:** 10.7150/ijbs.63390

**Published:** 2021-10-22

**Authors:** Lingling Jiang, Haiyi Fei, Xiaoying Jin, Xiu Liu, Cuiyu Yang, Chao Li, Jianmin Chen, Anran Yang, Jiajuan Zhu, Huihong Wang, Xiaoyang Fei, Songying Zhang

**Affiliations:** 1 Assisted Reproduction Unit, Department of Obstetrics and Gynecology, Sir Run Run Shaw Hospital, Zhejiang University School of Medicine, 310016, Hangzhou, China; 2 Department of Obstetrics and Gynecology, Key Laboratory of Reproductive Dysfunction, Management of Zhejiang Province, 310016, Hangzhou, China; 3 Department of Medical, Jiaxing University Affiliated Women and Children Hospital, 314051, Jiaxing, China; 4 Department of Obstetrics and Gynecology, Hangzhou Women's Hospital, 310008, Hangzhou, China

**Keywords:** Extracellular Vesicles, decidual NK Cells, secretion, HLA-E, pregnancy

## Abstract

Extracellular vesicles derived from trophoblasts (T-EVs) play an important role in pregnancy, but the mechanism is not entirely clear. In this study, we found that HLA-E, which is mostly confined to the cytoplasm of trophoblast cells, was secreted by T-EVs. The level of HLA-E in T-EVs from unexplained recurrent spontaneous abortion (URSA) patients was lower than that in normal pregnancy (NP) and RSA patients who had an abnormal embryo karyotype (AK-RSA). T-EVs promoted secretion of IFN-γ and VEGFα by decidual NK (dNK) cells from URSA patients via HLA-E, VEGFα was necessary for angiogenesis and trophoblast growth, and IFN-γ inhibited Th17 induction. Glycolysis and oxidative phosphorylation (OxPhos) were involved in this process. Glycolysis but not OxPhos of dNK cells facilitated by T-EVs was dependent on mTORC1 activation. Inhibition of T-EV production *in vivo* increased the susceptibility of mice to embryo absorption, which was reversed by transferring exogenous T-EVs. T-EVs promoted secretion of IFN-γ and VEGFα by dNK cells to maintain pregnancy via Qa-1 in abortion-prone mouse models. This study reveals a new mechanism of pregnancy maintenance mediated by HLA-E via T-EVs.

## Introduction

The embryo is a natural allograft to the maternal immune system. Developmentally and physiologically coordinated interactions between an embryo and maternal immune cells are critical for a successful pregnancy 1. The breakdown of coordination at the maternal-fetal interface can result in poor pregnancy outcomes, such as recurrent spontaneous abortion (RSA), which is characterized by the loss of two or more consecutive pregnancies and occurs in up to 5% of reproductively active couples 2,3. Approximately half of all cases remain unexplained, which are called unexplained RSA (URSA) 4. The cause is elusive or multifactorial, and misinformation abounds, giving rise to frustration for affected couples and their physicians 5.

During the first trimester of pregnancy, CD56^bright^ decidual natural killer (dNK) cells accumulate and become the dominant leukocyte population, constituting >70% of all leukocytes in the human decidualizing endometrium 6-8. In contrast to peripheral NK cells, dNK cells are unable to kill semiallogeneic fetal cells, and the main function of CD56^bright^ dNK cells in general may be to secrete cytokines, including growth-promoting factors (GPFs) 9, VEGFα 10 and IFN-γ 11,12, in early pregnancy to promote placental vascular growth, spiral artery remodeling, decidualization and immune balance. Interactions of NK cell-specific receptors with their ligands expressed on either invasive decidual stromal cells or trophoblast cells modulate the secretion of GPFs, VEGFα and IFN-γ by dNK cells 9,10,13. HLA-E, which is the ligand of CD94/NKG2A and CD94/NKG2C heterodimers expressed on dNK cells 14, may play an important role in regulating NK cells during pregnancy 15. However, HLA-E expression seems to be limited to the cytoplasm of trophoblast cells 16. We also found that HLA-E was mostly expressed in the cytoplasm rather than on the cell surface of trophoblast cells in this study. There is no clear evidence indicating how HLA-E regulates dNK cells and whether HLA-E is involved in the pathogenesis of RSA.

Emerging evidence has demonstrated that cellular metabolism is integral to NK cell effector functions 17. IL-15 stimulation-induced increases in glycolytic and respiration rates play an important role in ERK activation and NK cell expansion 18. Severe cellular metabolic deficiency impairs NK cell cytotoxicity, which can be reversed by metabolic reprogramming 19. NKG2C^+^ adaptive NK cells, which are associated with improved IFN-γ production and antiviral activity, exhibit enhanced oxidative and glycolytic metabolic profiles compared to NKG2C^-^ canonical NK cells 20, and elevated levels of oxidative phosphorylation (OxPhos) are required to support both cytotoxicity and IFN-γ production in both NKG2C^+^ NK cells and NKG2C^-^ NK cells 21. mTOR kinase has been reported to play a crucial role as a key metabolic checkpoint in NK cell proliferation, maturation and activation 22. It has been indicated that mTORC1 activity is essential for attaining the elevated glycolytic activity needed to support IFN-γ production and antitumor cytotoxicity in NK cells 19,23. However, how the distinct metabolic profiles of dNK cells during pregnancy drive NK cell functional fates is not yet well understood.

Extracellular vesicles with lipid bilayer structures, as mediators of communication between cells, have been the subject of increased focus 24. EVs consist of exosomes and microvesicles, which originate from the endosomal system and are shed from the plasma membrane, respectively 24. Exosomes are 40- to 200-nm vesicles released by the fusion of multivesicular bodies with the plasma membrane 25. During normal pregnancy, the number of EVs derived from the placenta present in the maternal plasma increases significantly through the first trimester 26, and the content and effect of placental EVs may be regulated by oxygen tension and blood glucose levels 27,28. Trophoblast-derived EVs (T-EVs) seem to generate pleiotropic effects on the maternal-fetal interface and maternal immune system during pregnancy 29. For example, human placenta-derived EVs bearing NKG2D ligands downregulate the NKG2D receptors on peripheral NK, CD8^+^, and γδ T cells, leading to reductions in cell cytotoxicity 30. Several studies suggest that placental EVs are capable of promoting cell migration and angiogenesis *in vitro* 28,31. However, how T-EVs interact with and regulate the phenotype and function of dNK cells remains to be elucidated.

In this study, we found that HLA-E was secreted by trophoblast cells via EVs. T-EVs promoted VEGFα and IFN-γ secretion by dNK cells by facilitating glycolysis and OxPhos via HLA-E, and OxPhos might involve mTORC1. In addition, we confirmed that T-EVs regulate dNK cells to maintain pregnancy via Qa-1 (HLA-E homolog in mice) *in vivo*. Therefore, our results reveal a still-unknown mechanism of the maintenance of pregnancy mediated by HLA-E via T-EVs and demonstrate the potential of T-EVs as biologic agents for the treatment of URSA.

## Materials and methods

### Human samples

Decidual and villus samples from normal pregnancies (n = 24) were obtained from patients who underwent elective pregnancy termination. Thirty-four decidual samples from abnormal pregnancies were obtained from patients with RSA, among which 16 samples had an abnormal karyotype (AK-RSA) and 17 samples had a normal karyotype (URSA). Anatomical causes of abortion were excluded. The normal and abnormal samples were aged between 6 and 8 weeks of gestation (Table S1). All of the decidual and villus samples were collected from the Sir Run Run Shaw Hospital, Zhejiang University School of Medicine. All subjects provided written informed consent for the collection and study of their samples. Ethical approval was obtained from the ethics committee of Sir Run Run Shaw Hospital, Zhejiang University School of Medicine.

### Mice and cell lines

Male DBA/2 and female CBA/J mice (8 to 10 weeks old) were purchased from Beijing HFK Bioscience Co., Ltd. Male Balb/c mice (8 to 10 weeks old) were purchased from the Shanghai Jihui Experimental Animal Breeding Co., Ltd. All animals were kept under specific pathogen-free conditions. All of the experimental procedures involving animals were conducted in accordance with the Guide for the Care and Use of Laboratory Animals (China), and the protocols were approved by the Animal Research Ethics Committee of Sir Run Run Shaw Hospital of Zhejiang University. JEG-3 cells, HTR-8/Svneo cells and HUVECs were obtained from the American Type Culture Collection (Manassas, VA, USA).

### Isolation of human dNK cells

For the isolation of human dNK cells, fresh decidual tissues were washed with cold sterile PBS twice, cut into small pieces and digested with collagenase type IV (1 mg mL-1; Sigma-Aldrich, St. Louis, MO, USA) and DNase I (0.01 mg mL-1; Sigma-Aldrich) in RPMI 1640 medium (Thermo Fisher Scientific, Waltham, MA, USA) for 40 min at 200 rpm and 37 °C. The suspensions were strained through 70-μm nylon mesh and then loaded onto a Ficoll density gradient to purify the lymphocytes. NK cells used in flow cytometry, TEM, immunofluorescence and metabolic assays were purified using the NK Cell Isolation Kit (Miltenyi Biotec, Bergisch Gladbach, Germany) 9.

### Human placental villous explants, culture, and incubation with dNK cells *in vitro*

Human placental villi were obtained from electively terminated pregnancies. The procedures used for the preparation of human placental villous explants have been previously reported 32,33. Briefly, chorionic villi dissected from placentas at 6 to 8 weeks of gestational age were cultured on explant medium consisting of Dulbecco's modified Eagle's medium/F12 (1:1) (Gibco, Carlsbad, CA) with 10% HyClone fetal bovine serum (FBS; Thermo Scientific), 1% penicillin-streptomycin, and 1% amino acid with DMSO or N-SMase spiroepoxide inhibitor (sc-202721; Santa Cruz Biotechnology, Santa Cruz, USA). After 24 hours, the explants were cocultured with dNK cells for 24 hours, and the intracellular expression of IFN-γ and VEGFα in dNK cells was detected by flow cytometry (FCM).

### T-EV isolation and characterization

EV isolation from tissue was conducted following published protocols 34-36. Briefly, for T-EV isolation, chorionic villi dissected from placentas at 6 to 8 weeks of gestational age were cut into small pieces in RPMI 1640 medium (Thermo Fisher Scientific). The suspensions were strained through 70-μm nylon mesh and then centrifuged at 3,000 ×g for 15 min to remove cells and cell debris. The supernatants were collected and filtered using a 0.22-μm filter and then centrifuged at 100,000×g for 1h at 4 °C. The EV pellets were washed in 25 mL of sterile PBS and centrifuged at 100,000×g for an additional 1 h. The final pellets were resuspended in PBS and stored at -80 °C. The amount of EV protein recovered was measured by a BCA assay (Thermo Fisher Scientific).

### Transmission electron microscopy (TEM)

dNK cells were purified by MACS (NK Cell Isolation Kit, human; Miltenyi Biotec), fixed with 2.5% glutaraldehyde at 4 °C for 12 hours, and then fixed in 2% osmium tetroxide. After adequate washing, the samples were stained with 1% aqueous uranyl acetate. Then, the samples were dehydrated with sequential washes in 50, 70, 90, 95 and 100% ethanol and immersed in Eponate 12 Resin. The samples were then cut into ultrathin sections and counterstained with uranyl acetate and lead citrate. Images were acquired with a Tecnai T10 100 kV electron microscope (Philips Medical Systems, Best, The Netherlansds) 37. The number of mitochondria in dNK cells in different groups was counted. A 3.5 μL T-EV sample was placed onto a carbon grid and allowed to rest for 60 s before blotting with filter paper. Using forceps, the carbon grid was dipped face-down into a water droplet for 2-3 s and blotted again. Aqueous uranyl acetate (1%, 3.5 μL) was pipetted onto the grid and allowed to rest for 15 s before blotting and drying. Images were acquired with a Tecnai G2 Spirit 120 kV (Thermo FEI, Thermo Fisher Scientific).

### Nanoparticle tracking analysis

The size and diameter of EVs were determined by nanoparticle tracking analysis (NTA) with a NanoSight NS300 (Malvern, USA). Isolated samples were appropriately diluted with PBS. NTA measurements were recorded and analyzed at 11 positions. A ZetaView system was calibrated using 110-nm polystyrene particles. The temperature was maintained at approximately 25 °C.

### Flow cytometry

dNK cells were purified using MACS (NK Cell Isolation Kit, human; Miltenyi Biotec). Suspensions of dNK cells were stained with the following human monoclonal antibodies (mAbs) and reagents: anti-CD3 (300420; BioLegend, San Diego, CA, USA), anti-CD56 (17-0567-42; Invitrogen, Thermo Fisher Scientific), 7AAD (MeilunBio, Dalian, China), anti-NKG2C (134591; Bio-Techne, Minnesota, USA), and anti-NKG2A (131411; Biotechne). Homologous IgGs were used as negative controls. dNK cells were cultured in complete RPMI 1640 medium (Gibco) with 10% fetal bovine serum (HyClone, Thermo Fisher Scientific) plus 1% streptomycin and penicillin and 50 ng/mL IL-15 at 37 °C for 72 hours. A cell stimulation cocktail (00-4970-03; Invitrogen) was added to dNK cells 12 hours before the cells were collected to detect intracellular cytokines. After surface staining with anti-CD3 (300420; BioLegend) and anti-CD56 (17-0567-42; Invitrogen) antibodies, the dNK cells were treated with IC fixation buffer (Invitrogen) and incubated with anti-VEGFα (ab52917; Abcam, Cambridge, UK) or anti-IFN-γ (ab9657; Abcam) as the primary antibody and anti-rabbit 488 (2156517; Invitrogen) or anti-mouse 549 (GAM5492; MULTI SCIENCES, Hangzhou, China) as the secondary antibody. FCM staining was performed according to the manufacturer's instructions, and data from 20,000-50,000 single-cell events were collected using a cytoFLEX flow cytometer (Beckman Coulter, Brea, CA, USA).

### Enzyme-linked immunosorbent assay (ELISA)

The concentrations of IFN-γ and VEGFα in cell culture supernatants were quantified using human IFN-γ and VEGFα ELISA kits (Thermo Fisher Scientific), respectively. ELISAs were performed according to the manufacturer's instructions.

### Western blotting and FCM analyses of T-EVs

For western blotting detection, a total of 30 μg of T-EVs or crude protein extracted from cell lysates was separated by 12% SDS-PAGE and transferred to a polyvinylidene difluoride membrane (Millipore, Danvers, Massachusetts, USA). The membranes were blocked with 5% BSA in TBST and then incubated with appropriate primary antibodies overnight at 4 °C. After incubation with horseradish peroxidase-coupled secondary antibodies for 1 h, the membranes were scanned using a ChemiDoc MP Chemiluminescence imager (Bio-Rad, Hercules, California, USA) according to the manufacturer's instructions. For FCM analysis, 20 μg of EVs was incubated with 5 μL of 4-μm-diameter aldehyde/sulfate latex beads (Invitrogen) for 15 min at room temperature in PBS, with a final volume of 20 μL. The mixture was then transferred to 1 mL of PBS and incubated with gentle shaking for 1 h. After centrifugation, the pellet was blocked by incubation with 20 μL of fetal bovine serum for 30 min. EV-coated beads were washed three times in PBS and resuspended in 50 μL of PBS. Afterward, the beads were incubated with appropriate fluorophore-conjugated antibodies for 1 h at room temperature in the dark. The beads were analyzed by FCM (Beckman Coulter) 38.

### Confocal microscopy

Decidual tissues were fixed with 4% PFA at 4 °C overnight, incubated in 30% sucrose for 24h at 4 °C, coated with embedding medium and finally snap frozen. Purified dNK cells and cryostat sections were fixed with 4% PFA and incubated in blocking buffer (5% normal goat serum and 0.5% Triton-X in PBS) at room temperature for 1 hour. Primary antibodies against CD56 (1:100; 3576; Cell Signaling Technology, Danvers, Massachusetts, USA) and HLA-E (1:100; 2216; Abcam) and secondary antibodies (anti-rabbit 488 (2156517; Invitrogen) or anti-mouse 549 (GAM5492; MULTI SCIENCES)) were added, followed by staining with DAPI and acquisition on a Zeiss LSM800 confocal laser scanning microscope. All immunofluorescence staining was performed in the dark. For mitochondrial imaging, freshly isolated dNK cells were stained with MitoTracker Red (Invitrogen) for 30 min, and then the above steps were performed. The average fluorescence intensity of each cell was calculated using ImageJ.

### qRT-PCR

Whole DNA was extracted using the Genomic DNA Easy Mini Kit (Life Science, Thermo Fisher Scientific), and qRT-PCR was performed using SYBR Green PCR Master Mix (Applied Biosystems, Thermo Fisher Scientific). The GAPDH gene was used to estimate nuclear DNA (nDNA) levels, and the ATP synthase (ATPase) 8 mitochondrial DNA gene was used to estimate mitochondrial DNA (mtDNA) levels. The primers used for qRT-PCR analysis of GAPDH were forward: 5'-CCCCACACACATGCACTTACC-3' and reverse: 5'-CCTAGTCCCAGGGCTTTGATT-3', while those used for ATPase 8 were forward: 5'-AATATTAAACACAAACTACCACCTACC-3' and reverse: 5'-TGGTTCTCAGGGTTTGTT ATA-3' 39.

### Tube formation assay

A 96-well plate was prepared by adding 50 μL of Growth Factor Reduced Matrigel (10 mg/mL, BD Biosciences, Franklin Lakes, NJ, USA) and incubating the plate overnight at 37 °C under normoxic conditions to form a semisolid gel-like matrix. HUVECs were resuspended in serum-free RPMI 1640 medium and seeded (1x10^4^ cells/well) into individual wells containing different dNK cell supernatants in a total volume of 200 µl. Sixteen hours later, the HUVECs were viewed under a fluorescence microscope (Carl Zeiss, Oberkochen, Germany), and angiogenesis in the different groups was calculated with ImageJ 40.

### *In vivo* tumor growth assay

Eight-week-old nude mice (5-6 mice per group) were subcutaneously inoculated with JEG-3 cells (2x10^6^ cells in 200 µl of PBS). Fourteen days later, when the tumors became measurable, the mice were injected with supernatants obtained from different groups of dNK cells. The tumor size was determined with calipers on the next five days, and another injection of supernatants was given immediately after the first measurement. In a blocking experiment, an anti-VEGFα antibody (52917; Abcam) was added at a concentration of 200 ng per mL to the supernatants containing 20 ng per mL VEGFα, followed by incubation for 2 hours at 37 °C before injection 10.

### OCR and ECAR measurement

For measurement of the OCR and ECAR of dNK cells, freshly isolated cells (200,000 cells/well) were treated with or without rapamycin (10 nM) for 2 h and then incubated with or without T-EVs or anti-HLA-E blocking antibody-treated T-EVs in RPMI 1640 medium containing 50 ng/mL hIL-15 overnight. Then, the cells were plated on polylysine-pretreated Seahorse plates in XF medium (25 mM glucose, 2 mM glutamine and 1 mM pyruvate) and analyzed using an XF-8 Extracellular Flux Analyzer (Agilent Technologies, Santa Clara, California, USA). The cells were treated with 2 mM oligomycin, 1.5 mM FCCP, 1 mM rotenone and 1 µM antimycin A (all drugs were from Agilent Technologies) to measure the OCR. For measurement of the ECAR, glucose (10 mM), oligomycin A (1 mM) and 2-DG (50 mM) were added 37,41.

### Animal experiment

Eight-week-old female ICR mice were mated with 10-week-old male ICR mice, and the detection of a vaginal plug was chosen to identify day 0.5 of gestation 42. ICR females received three intrauterine perfusions with DMSO or 50 µM spiroepoxide on days 3.5, 6.5 and 9.5 and three tail vein injections with PBS or 100 μg T-EVs on days 4.5, 7.5 and 10.5. The mice were euthanized on day 14.5, and the uteri and fetuses were obtained. The percentage of resorbed embryos was calculated, and intracellular expression of IFN-γ and VEGFα in NK1.1^+^ NK cells in the uteri was detected by FCM.

In the control group, female CBA/J mice were mated with male Balb/c mice in natural cycles, and in the abortion-prone group, female CBA/J mice were mated with male DBA/2 mice in natural cycles. The detection of a vaginal plug was used to identify day 0.5 of gestation. Pregnant female mice received three injections of 200 µg of mouse T-EVs intravenously on days 1.5, 3.5 and 5.5 via the tail vein. All of the mice were euthanized at 14.5 days of gestation to examine the fetal resorption rate, Qa-1 level of T-EVs from the placenta and cytokine expression of dNK cells. The percentage of resorbed embryos was calculated as follows: resorbed embryos/total embryos x 100. Uteri from pregnant mice were dissected, washed twice in ice-cold PBS and cut into small pieces. The minced uteri were enzymatically digested with collagenase type IV (1 mg mL^-1^; Sigma-Aldrich, St. Louis, MO, USA) and DNase I (0.01 mg mL^-1^; Sigma-Aldrich) in RPMI 1640 medium (Thermo Fisher Scientific) for 40 min at 200 rpm and 37 °C. The suspensions were strained through 70-μm nylon mesh and then subjected to flow cytometry after surface staining for NK1.1 and intracellular staining for VEGFα and IFN-γ. The placenta were dissected free from the uteri and subjected to the isolation of EVs following the method used for human T-EV isolation.

### Statistical analysis

Statistical significance was determined using Prism 6.0 (GraphPad version 6). Comparisons between two groups were performed using Student's t-test, and comparisons among multiple groups were performed using one-way analysis of variance (ANOVA). The Spearman rank-order correlation test was used to examine correlations between the HLA-E level of T-EVs and IFN-γ or VEGFα expression in dNK cells from RSA patients and healthy people. All of the data are presented as the mean ± SEM (*, *P* < 0.05, **, *P* < 0.01, ***, *P* < 0.001, and ****, *P* < 0.0001).

## Results

### HLA-E confined to the cytoplasm of trophoblast cells was secreted via EVs in early pregnancy

**Figure 1:**
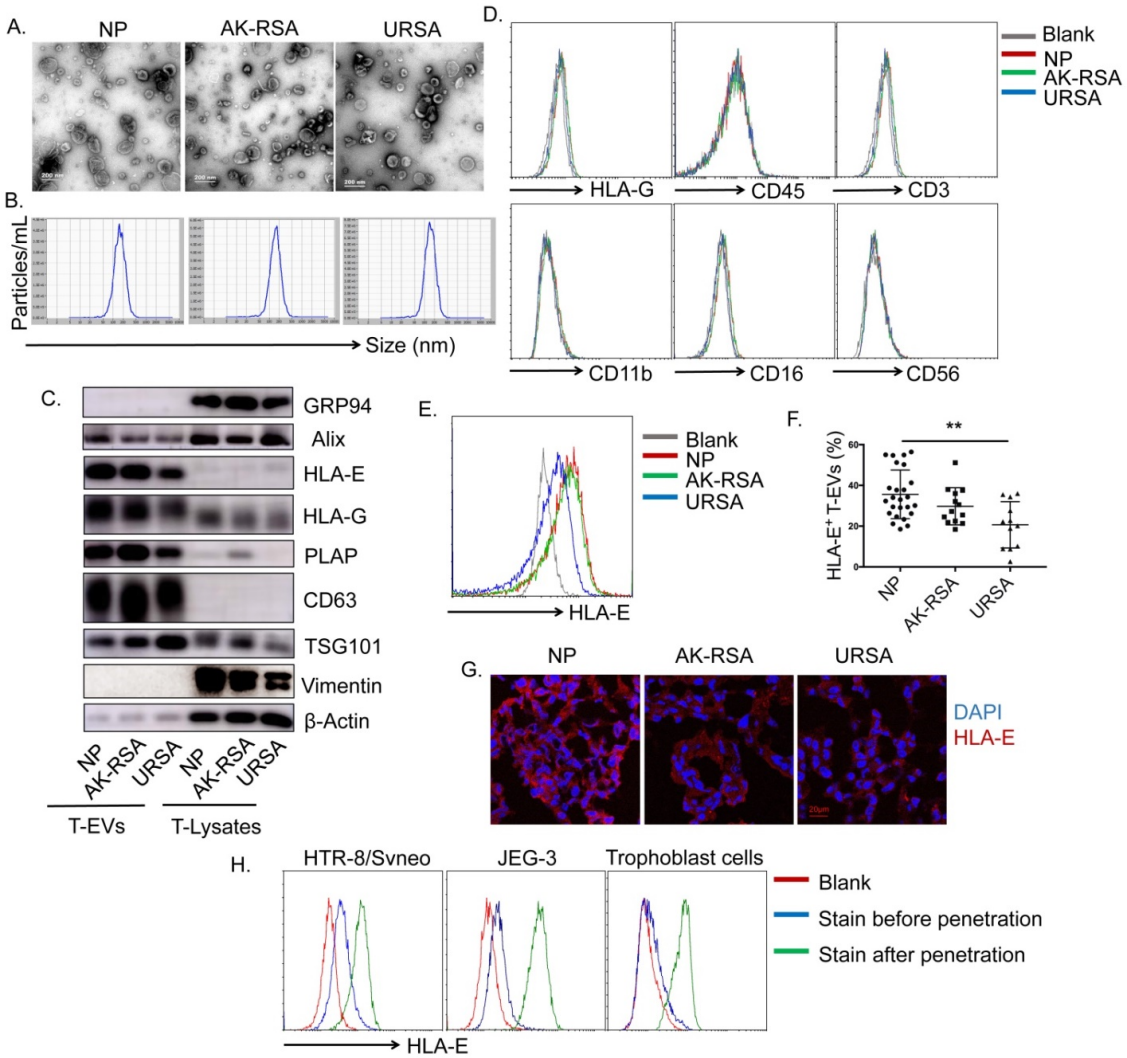
** Characterization of trophoblast-derived EVs (T-EVs).** EVs were isolated from the villi according to a standard isolation procedure. **A.** Representative electron micrograph of T-EVs from patients with a normal pregnancy (NP), RSA patients who had an abnormal embryo karyotype (AK-RSA) and URSA patients.** B.** Nanoparticle tracking analysis (NTA) of T-EVs from NP, AK-RSA and URSA patients. **C.** A total of 30 μg of T-EVs and total villus lysates were immunoblotted with the indicated antibodies. **D.** After adsorption onto latex beads, T-EVs from NP, AK-RSA and URSA patients were phenotyped and analyzed by FCM with the indicated antibodies. **E.** After adsorption onto latex beads, T-EVs from NP, AK-RSA and URSA patients were analyzed by FCM with an anti-HLA-E PE-conjugated mAb (gray: isotype control; red: NP; green: AK-RSA; blue: URSA). **F.** The percentage of HLA-E^+^ beads was statistically analyzed (n=25 in the NP group; n=13 in the AK-RSA patient group; n=12 in the URSA patient group). **G.** The expression of HLA-E in villus tissues from NP, AK-RSA and URSA patients was detected by an immunofluorescence assay. **H.** Human villus trophoblast cells, JEG-3 cells and HTR-8/Svneo cells were analyzed by FCM with an anti-HLA-E PE-conjugated mAb. Red: isotype control; blue: stained before permeabilization with IC fixation buffer (Invitrogen); green: stained after permeabilization with IC fixation buffer (Invitrogen). The data are representative of three independent experiments.

T-EVs were isolated from patients with a normal pregnancy (NP), RSA patients who had an abnormal embryo karyotype (AK-RSA) and URSA patients *in vitro*. EVs in the three groups ranged in size from 40-200 nm (Figure 1A). Size distribution analysis revealed that the mean sizes of the T-EVs from the patients with NP, AK-RSA or URSA were 154.7, 151.9 and 145.8 nm with Z-potentials of -33.88, -38.04 and -37.29 mV, respectively (Figure 1B). The three groups of T-EVs were all positive for CD63, Alix and TSG101 molecules but were negative for the endoplasmic reticulum-residing protein GRP94 and mesenchymal cell-specific molecule vimentin and rich in HLA-E, HLA-G and PLAP (Figure 1C). In addition, the T-EVs were HLA-G-, CD45-, CD11b-, CD56-, CD3- and CD16-negative on the surface (Figure 1D). These data suggested the derivation of these EVs from trophoblast cells rather than immune cells in the villi.

We found that the T-EVs from URSA patients contained lower levels of HLA-E than those from patients with NP or AK-RSA; there was no significant difference in HLA-E levels between T-EVs from patients with NP and AK-RSA patients (Figure 1C, E and F). We found that HLA-E was localized to the syncytiotrophoblast, villous mesenchymal cells and extravillous trophoblast (EVT), but staining for HLA-E appeared to be confined primarily to the cytoplasm (Figure 1G). In addition, surface staining for HLA-E on human villus trophoblast cells and the JEG-3 and HTR-8/Svneo cell lines using flow cytometry (FCM) was faint; however, cytoplasmic staining for HLA-E in these cells after permeabilization was remarkably positive (Figure 1H). These data revealed that HLA-E was mostly expressed in the cytoplasm rather than on the surface of trophoblast cells. This suggests that HLA-E may leave the cell membrane to function through loading onto EVs.

### T-EVs promoted the secretion of IFN-γ and VEGFα by dNK cells from URSA donors via HLA-E

**Figure 2:**
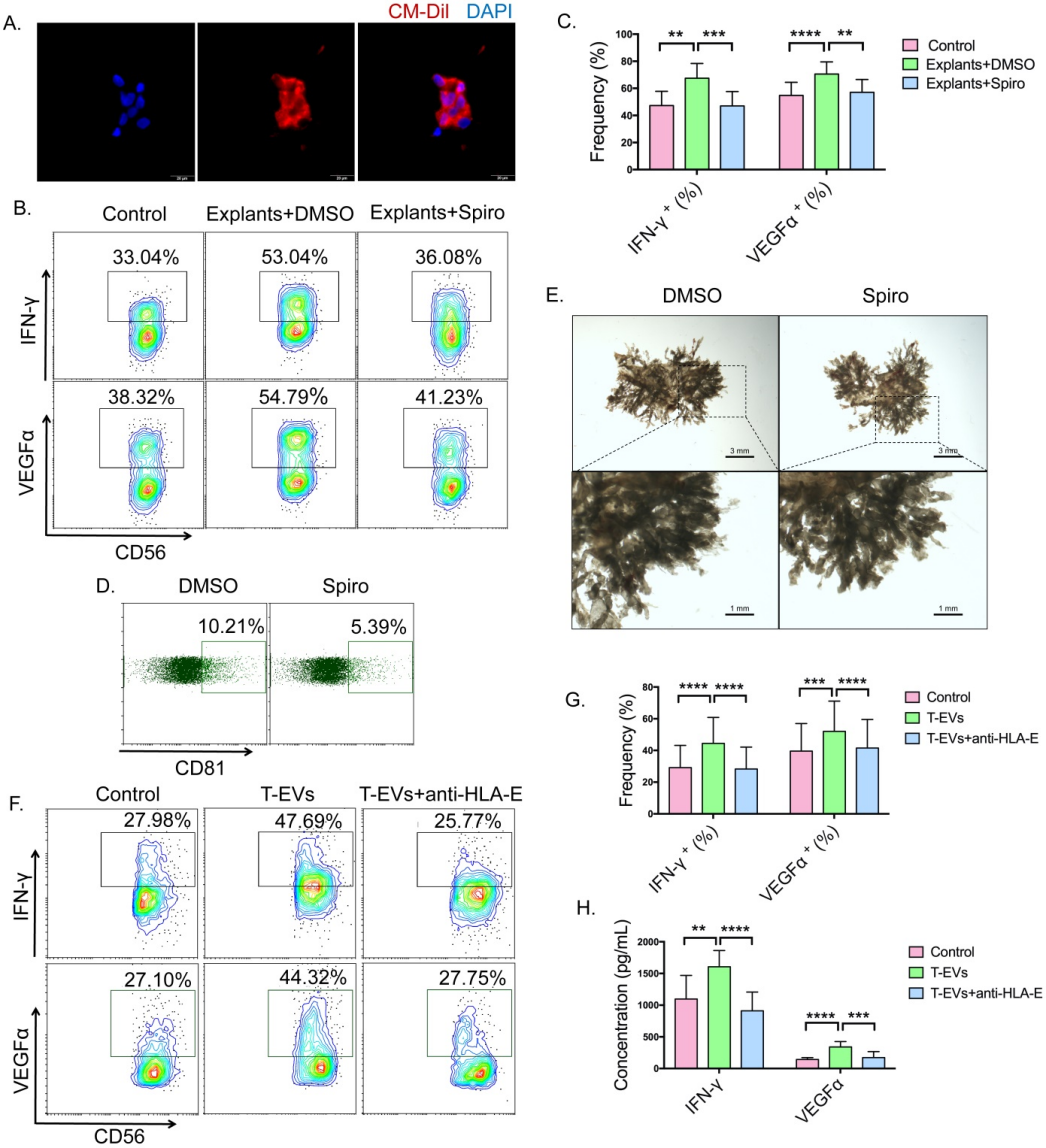
** T-EVs promoted the secretion of IFN-γ and VEGFα by dNK cells from URSA donors via HLA-E *in vitro*. A.** CM-Dil-labeled T-EVs were added at a concentration of 50 µg/mL to a dNK cell culture system. The nuclei of dNK cells from URSA patients were stained using DAPI, and an immunofluorescence assay was performed. **B.** Chorionic villi dissected from placentas at 6 to 8 weeks of gestational age were cultured with DMSO or N-SMase spiroepoxide inhibitor. After 24 hours, the explants were cocultured with dNK cells for 24 hours, and the intracellular expression of IFN-γ and VEGFα in dNK cells was detected by FCM.** C.** The intracellular expression of IFN-γ and VEGFα in dNK cells detected using FCM was statistically analyzed. **D.** The EVs in the supernatants of explants cultured with DMSO or N-SMase spiroepoxide inhibitor in **B.** were adsorbed onto CD63-coated latex beads, and the percentage of CD81-positive latex beads was detected by FCM. **E.** Representative micrograph of the explants cultured with DMSO or N-SMase spiroepoxide inhibitor. **F, G.** T-EVs with or without anti-HLA-E blocking antibody treatment were incubated with dNK cells from URSA patients at a concentration of 50 µg/mL in the dNK cell culture system. The intracellular expression of IFN-γ and VEGFα in dNK cells from URSA patients was detected by FCM and statistically analyzed. **H.** Supernatants of the dNK cells in **F.** were detected by ELISA, and the results were statistically analyzed. *P* values were generated by one-way analysis of variance (ANOVA) followed by the Newman-Keuls multiple comparison test using GraphPad Prism 6 (n=14, *P < 0.05, ** *P* < 0.01, *** *P* < 0.001, **** *P* < 0.0001, NS, not significant).

Because T-EVs contain high levels of HLA-E, we examined the effect of T-EVs on dNK cells, which compose the major leukocyte subpopulation in the human decidua and express receptors for HLA-E, CD94/NKG2 13,43. dNK cells display decreased cytotoxicity and have been shown to be the main cytokine-producing NK cells, secreting cytokines such as IFN-γ and VEGFα, which might be important in spiral artery remodeling, angiogenesis and the development of the placenta 10,11. Firstly, CM-Dil-labeled T-EVs fused with the cell membrane to allow continuity with the cytoplasm of dNK cells (Figure 2A), suggesting that T-EVs can interact with dNK cells *in vitro*. We cultured human villous explants with or without spiroepoxide, which has been reported to inhibit EV release 38,44. Then, purified dNK cells were cocultured with the villous explants, and the levels of IFN-γ and VEGFα were detected. The secretion of IFN-γ and VEGFα by dNK cells increased after incubation with villous explants, and inhibition of the endogenous production of EVs by trophoblast cells decreased the secretion of IFN-γ and VEGFα by dNK cells (Figure 2B and C). We confirmed that spiroepoxide inhibited T-EV release from human villous explants (Figure 2D) but did not affect the growth of villous explants *ex vivo* (Figure 2E). These data suggest that T-EVs may be involved in the secretion of IFN-γ and VEGFα by dNK cells.

Then, we measured IFN-γ and VEGFα levels in dNK cells from NP, AK-RSA and URSA donors using flow cytometry (FCM). Our results indicated that the surface expression of NKG2C and the intracellular expression of IFN-γ and VEGFα in CD3^-^CD56^+^ dNK cells from URSA patients (Figure S1A) were significantly lower than those from patients with NP and AK-RSA patients (Figure S1B-D), and the decreased secretion of VEGFα and IFN-γ by dNK cells from URSA patients was also confirmed by ELISA (Figure S1E).

By analyzing the correlations between the HLA-E levels in T-EVs and IFN-γ and VEGFα levels in dNK cells, we found that the HLA-E levels in T-EVs were positively correlated with the IFN-γ and VEGFα levels in dNK cells (Figure S1F), demonstrating that the IFN-γ and VEGFα levels in dNK cells were decreased in URSA patients, probably in an HLA-E-dependent manner.

To further confirm the effect of T-EVs on dNK cells, purified dNK cells from URSA patients were incubated with T-EVs *in vitro*. Intracellular staining showed that T-EVs facilitated intracellular IFN-γ and VEGFα (Figure 2F and G) expression in dNK cells from URSA patients, and increased secretion of IFN-γ and VEGFα by dNK cells was confirmed by ELISA (Figure 2H). To elucidate the role of HLA-E in the activation potential of T-EVs, we pretreated T-EVs with an anti-HLA-E blocking antibody as a potent and selective inhibitor of HLA-E binding with the CD94/NKG2 receptor. After the blockage of HLA-E, the T-EVs hardly promoted IFN-γ or VEGFα expression in dNK cells from URSA patients (Figure 2F and G). In addition, the amounts of IFN-γ and VEGFα secreted by the dNK cells into the supernatant were quantified, which confirmed that HLA-E blockade prevented the promotion of the secretion of IFN-γ and VEGFα in dNK cells induced by T-EVs (Figure 2H).

To confirm that T-EVs affect the secretion of IFN-γ and VEGFα in dNK cells via HLA-E, we knocked down HLA-E expression in JEG-3 cells, which are a human chorionic cell line, via lentiviral vector delivery of HLA-E-specific siRNA and collected EVs in the supernatant. We found that HLA-E levels were significantly decreased in EVs derived from HLA-E-specific siRNA-treated JEG-3 cells (Figure S2A). Unlike the EVs derived from JEG-3 cells treated with negative control siRNA, those derived from HLA-E-specific siRNA-treated JEG-3 cells hardly promoted IFN-γ or VEGFα expression in dNK cells from URSA patients (Figure S2B and C).

These data suggest that the secretion of IFN-γ and VEGFα by dNK cells is regulated by T-EVs under physiological conditions and that T-EVs promote the secretion of IFN-γ and VEGFα by dNK cells via HLA-E *in vitro*.

### T-EVs facilitated glycolysis and OxPhos to promote the secretion of IFN-γ and VEGFα by dNK cells from URSA donors via HLA-E

Several studies have revealed that a robust metabolic response required for normal effector function occurs in activated NK cells 45,46. To determine the importance of metabolism in the secretion of IFN-γ and VEGFα by dNK cells induced by T-EVs, dNK cells from patients with NP were cultured with the glucose metabolism inhibitor 2-deoxy-ᴅ-glucose (2-DG) or ATP synthase inhibitor oligomycin, and we found that both 2-DG and oligomycin partly blocked intracellular expression of VEGFα and IFN-γ in dNK cells (Figure S3A and B). The secretion of VEGFα and IFN-γ by dNK cells was confirmed by ELISA (Figure S3C). These data suggested that glycolysis and OxPhos might be involved in the secretion of IFN-γ and VEGFα by dNK cells.

**Figure 3:**
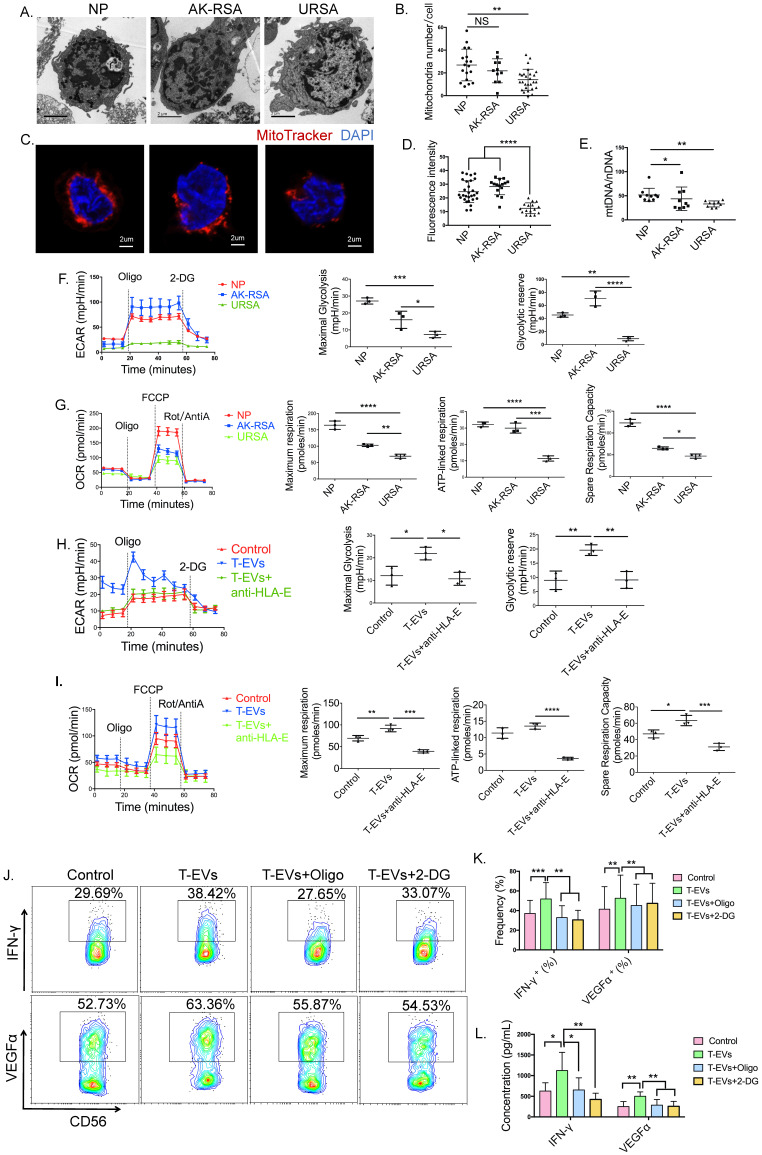
** T-EVs facilitated glycolysis and OxPhos to promote the secretion of IFN-γ and VEGFα by dNK cells from URSA donors via HLA-E. A.** Representative electron micrograph of mitochondria in purified dNK cells from NP, AK-RSA or URSA patients; scale bar = 2 µm. **B.** The number of mitochondria in** A.** was quantified and statistically analyzed. **C.** Confocal laser scanning microscopy images showing purified dNK cells in which the mitochondria (MitoTracker; red) and nucleus (DAPI; blue) were stained. **D.** Fluorescence intensity of the mitochondria, as analyzed by ImageJ. **E.** qRT-PCR was used to determine the ratio of mitochondrial DNA to genomic DNA for each indicated dNK cells. The results are from three independent replicates. **F.** ECAR profiles of purified dNK cells from NP, AK-RSA or URSA patients in a representative experiment and the average maximal glycolysis and glycolytic reserve. **G.** OCR profiles of purified dNK cells from NP, AK-RSA or URSA patients in a representative experiment and the average maximal respiration, ATP-linked respiration, and SRC. **H.** The ECAR profiles of purified dNK cells from URSA patients pretreated with or without T-EVs or T-EVs pretreated with an anti-HLA-E blocking antibody were evaluated. **I.** The OCR profiles of purified dNK cells from URSA patients pretreated with or without T-EVs or T-EVs pretreated with the anti-HLA-E blocking antibody were evaluated. The data are representative of three independent experiments or are shown as the mean ± s.e.m. pooled from three independent experiments. **J.** Intracellular expression of IFN-γ and VEGFα in dNK cells from URSA patients pretreated with or without the ATP synthase inhibitor oligomycin (20 µM) or glycolytic inhibitor 2-DG (1 mM) for 2 h before incubation with or without T-EVs was analyzed by FCM, and K. statistically analyzed. **L**. Supernatants of the dNK cells in J. were detected by ELISA, and the results were statistically analyzed. *P* values were generated by one-way analysis of variance (ANOVA) followed by the Newman-Keuls multiple comparison test using GraphPad Prism 6 (n=28 in the NP group; n=15 in the AK-RSA patient group; n=21 in the URSA patient group, *P < 0.05; ** P < 0.01; *** P < 0.001; NS, not significant).

It has been revealed that tumor-infiltrating NK cells in human liver cancers have small, fragmented mitochondria in their cytoplasm, which is correlated with reduced mitochondrial metabolism and limited NK cell-based tumor immunosurveillance 47. Since 2-DG and oligomycin partly blocked intracellular expression of VEGFα and IFN-γ in dNK cells, we hypothesized that dNK cells from URSA patients also had abnormal mitochondria. Transmission electron microscopy (TEM) revealed that the number of mitochondria in dNK cells from URSA patients was reduced compared to that in those from patients with NP or AK-RSA (Figure 3A and B). Similar changes in the number of mitochondria in dNK cells were observed by confocal laser scanning microscopy (CLSM) (Figure 3C and D). We confirmed decreased mitochondrial levels in dNK cells from URSA patients by quantification of the ratio of mitochondrial DNA to nuclear DNA by quantitative RT-PCR (qRT-PCR), and a remarkable decrease in the mitochondrial DNA ratio in dNK cells from URSA patients was observed (Figure 3E).

To directly analyze metabolic responses in dNK cells from distinct sources, we sorted dNK cells from NP, AK-RSA and URSA donors and performed Seahorse assays. In these experiments, we observed markedly attenuated extracellular acidification rate (ECAR) profiles for dNK cells from URSA patients, with significant reductions in maximal glycolysis and the glycolytic reserve (Figure 3F), and attenuated oxygen consumption rate (OCR) profiles for dNK cells from URSA patients, with significant reductions in maximal respiration, ATP-linked respiration, and spare respiration capacity (SRC) (Figure 3G). We examined the effect of T-EVs on the ECAR and OCR profiles of dNK cells from URSA patients *in vitro*. Seahorse assays indicated that T-EVs increased the maximal glycolysis, glycolytic reserve (Figure 3H), maximal respiration and SRC (Figure 3I) in dNK cells from URSA patients. To elucidate the role of HLA-E in the activation potential of T-EVs for the metabolism of dNK cells, we pretreated T-EVs with an anti-HLA-E blocking antibody. After the blockage of HLA-E, T-EVs hardly promoted glycolysis or OxPhos in dNK cells from URSA patients (Figure 3H and I). This result suggested that T-EVs facilitated glycolysis and OxPhos in dNK cells from URSA donors via HLA-E.

To confirm whether the promotion of the secretion of IFN-γ and VEGFα in dNK cells induced by T-EVs was dependent on metabolism, we cultured dNK cells from URSA patients with 2-DG, which blocks the first 2 enzymes of glycolysis, and demonstrated that 2-DG could partly block the VEGFα and IFN-γ secretion promoted by T-EVs (Figure 3J and K). In addition, we included oligomycin, which also partly abrogated the elevation of VEGFα and IFN-γ expression in dNK cells from URSA donors induced by T-EVs (Figure 3J and K). The secretion of VEGFα and IFN-γ by dNK cells was confirmed by ELISA (Figure 3L).

These data indicated that T-EVs might promote IFN-γ and VEGFα secretion by facilitating cellular metabolism in dNK cells via HLA-E.

### mTORC1 participated in the T-EV promotion of the secretion of IFN-γ and VEGFα by dNK cells by facilitating glycolysis but not OxPhos

**Figure 4:**
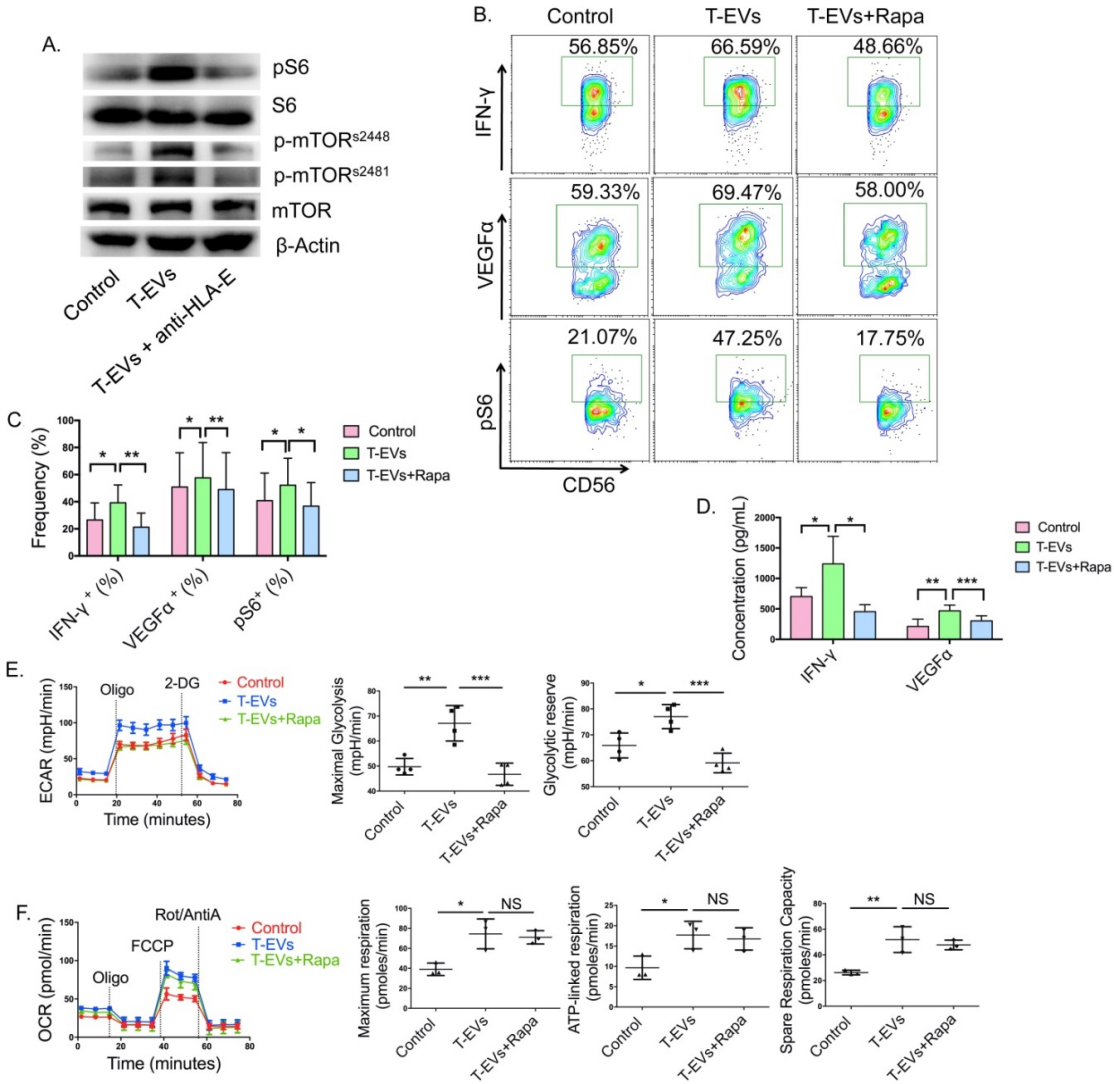
** mTORC1 participated in the T-EV promotion of the secretion of IFN-γ and VEGFα by dNK cells by facilitating glycolysis but not OxPhos. A.** Western blot analysis of pS6, S6, mTOR, p-mTOR^s2481^, and p-mTOR^s2448^ in dNK cells coincubated with or without T-EVs or T-EVs pretreated with an anti-HLA-E blocking antibody. **A** representative blot is shown. **B.** Intracellular staining for pS6, IFN-γ and VEGFα in dNK cells incubated with 50 µg/mL T-EVs with or without 10 nM mTORC1 inhibitor (rapamycin) was evaluated by FCM. **C.** The pS6, IFN-γ and VEGFα expression levels of dNK cells from URSA patients were statistically analyzed. **D.** Supernatants of the dNK cells in **B.** were detected by ELISA, and the results were statistically analyzed (n=6). **E.** ECAR profiles of purified dNK cells from URSA patients treated with 50 µg/mL T-EVs with or without 10 nM rapamycin in a representative experiment and the average maximal glycolysis and glycolytic reserve. **F.** OCR profiles of purified dNK cells from URSA patients treated with 50 µg/mL T-EVs with or without 10 nM rapamycin in a representative experiment and the average maximal respiration, ATP-linked respiration, and SRC.

We found that the metabolism of dNK cells was regulated by T-EVs. mTORC1 is a critical regulator of NK cellular metabolism 17, and it has been shown to facilitate glycolysis in CD56^bright^ NK cells 21. We found that T-EVs facilitated phosphorylated S6 (pS6) production and mTOR phosphorylation at positions 2448 and 2481 in dNK cells, and these effects were partly blocked by an anti-HLA-E blocking antibody (Figure 4A). To investigate whether T-EVs promote IFN-γ and VEGFα secretion and metabolism in dNK cells from URSA patients by activating mTORC1, the mTORC1 inhibitor rapamycin was used. mTORC1 activity in NK cells was determined by evaluating pS6, which is a downstream target of mTORC1 signaling. Rapamycin decreased the pS6 levels in dNK cells from NP and significantly decreased VEGFα and IFN-γ levels, suggesting that mTORC1 is active in dNK cells and might participate in VEGFα and IFN-γ secretion by these cells (Figure S3D and E). pS6 levels were substantially elevated in T-EV-treated dNK cells from URSA patients and were significantly inhibited by rapamycin (Figure 4B and C). The promotion of VEGFα and IFN-γ expression by dNK cells induced by T-EVs was reversed by the mTORC1 inhibitor rapamycin, demonstrating that T-EVs might promote the secretion of IFN-γ and VEGFα by dNK cells from URSA patients via mTORC1 (Figure 4B and C). The secretion of VEGFα and IFN-γ was confirmed by ELISA (Figure 4D). In addition, purified dNK cells were incubated with T-EVs overnight in the presence or absence of rapamycin before metabolic analysis. Rapamycin inhibited the increases in maximal glycolysis and the glycolytic reserve (Figure 4E) but not those in maximal respiration, ATP-linked respiration, or SRC induced by T-EVs (Figure 4F), demonstrating that mTORC1 participates in the process by which T-EVs promote glycolysis but not that by which they promote OxPhos in dNK cells from URSA patients.

Taken together, these results show that T-EVs facilitate VEGFα and IFN-γ secretion in dNK cells by promoting glycolysis but not OxPhos via mTORC1.

### T-EVs facilitate angiogenesis, trophoblast cell growth and inhibit Th17 induction by promoting the secretion of VEGFα and IFN-γ by dNK cells

**Figure 5:**
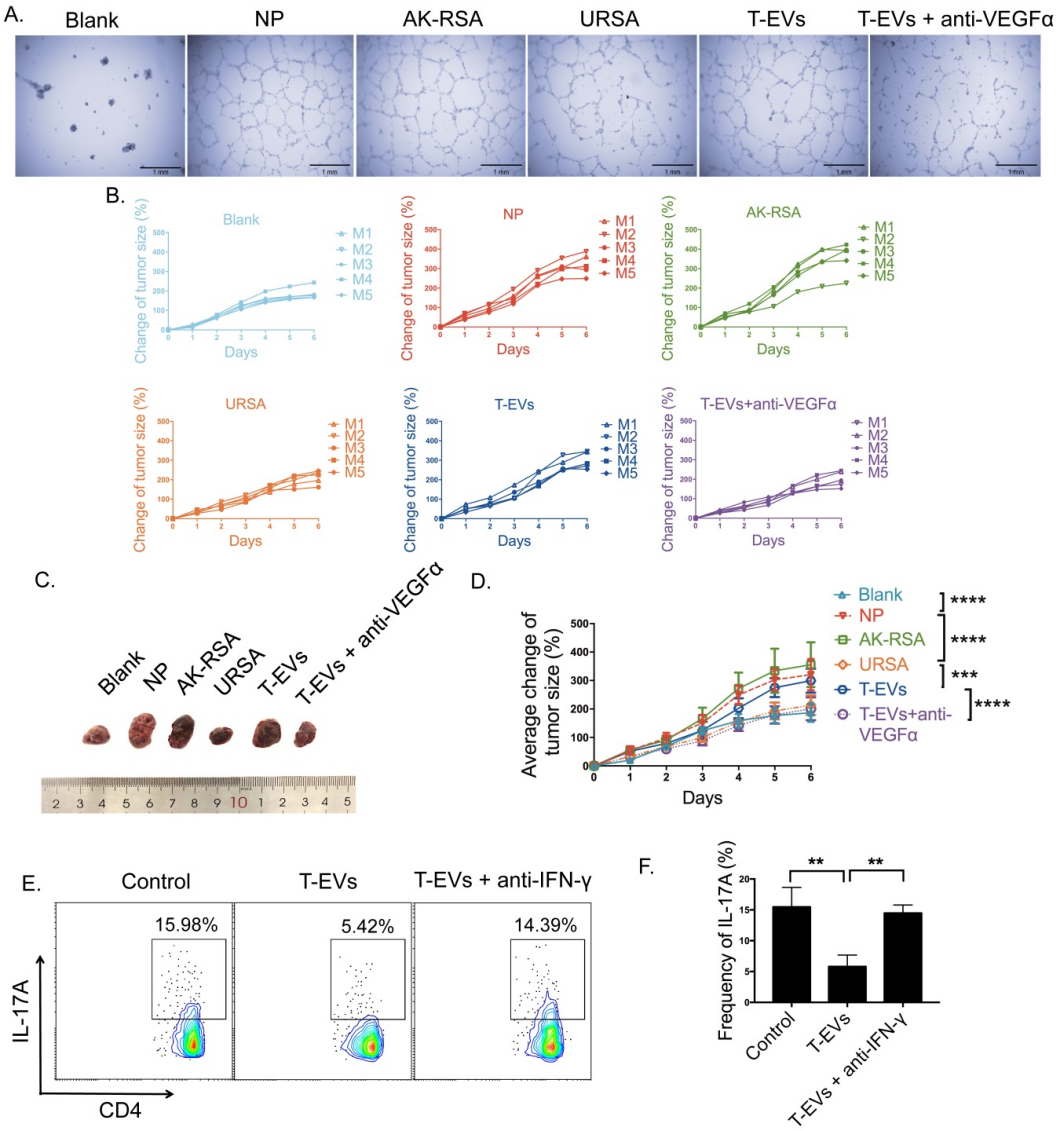
** T-EVs facilitated angiogenesis and trophoblast cell growth via VEGFα and inhibited Th17 induction via IFN-γ by dNK cells. A.** A tube formation test was performed on Growth Factor Reduced Matrigel. HUVECs were incubated with supernatants from purified dNK cells from NP, AK-RSA or URSA patients or supernatants from purified dNK cells from URSA patients in the presence of 50 µg/mL T-EVs. The incubations were performed with or without 200 ng/mL anti-VEGFα blocking antibody. **B.** JEG-3 cells were injected subcutaneously into nude mice. Fourteen days later, when the tumors became measurable, the mice were injected with supernatants from the different dNK cell groups described in **A.** (PBS, NP, AK-RSA, URSA, T-EVs, and T-EVs + anti-VEGFα, as indicated above each graph). The tumor size in each mouse was measured one day later (another injection of cell supernatants was given between the two measurements). **C.** Representative photographs of tumors from the different groups described in **B. D.** Summary of the changes in tumor size in each group over time (n=5, *** *P*<0.001, **** P < 0.0001). **E.** CD4^+^T cells isolated from peripheral blood mononuclear cells were cultured in complete RPMI 1640 medium with 10% fetal bovine serum (HyClone) plus 1% streptomycin and penicillin and 25 ng/mL IL-1β and 25 ng/mL IL-23, then incubated with supernatants from purified dNK cells from URSA patients treated with or without 50 µg/mL T-EVs. The incubation with supernatant from purified dNK cells from URSA patients treated with 50 µg/mL T-EVs were performed with or without 200 ng/mL anti-IFN-γ blocking antibody. Three days later, intracellular expression of IL-17A in CD4^+^ T cells was detected by FCM and **(F)** analyzed statistically.

A previous study confirmed that the activation of dNK cells through repeated pregnancies led to increased production of VEGFa, supporting vascular sprouting and tumor growth 10. To test the functionality of VEGFα secretion by dNK cells induced by T-EVs, we employed *in vitro* (Figure 5A) and *in vivo* (Figure 5B-D) models. Supernatants obtained from dNK cells from NP, AK-RSA or URSA donors and dNK cells from URSA donors incubated with T-EVs in the presence or absence of anti-VEGFα neutralizing antibodies were incubated with human umbilical vein endothelial cells (HUVECs), and limited tube formation was observed when phosphate-buffered saline (PBS) or URSA supernatants were used. In contrast, marked tube formation was observed with supernatants derived from NP donors and URSA donors incubated with T-EVs, and this effect was partially blocked when anti-VEGFα neutralizing antibodies were included in the assay (Figure 5A). We next tested the functionality of dNK cells from NP, AK-RSA and URSA donors in an *in vivo* model. We employed nude mice and injected them subcutaneously with choriocarcinoma-derived JEG-3 cells. When the tumors were palpable, we injected PBS or supernatants obtained from dNK cells from NP, AK-RSA or URSA donors or dNK cells from URSA donors incubated with T-EVs in the presence or absence of anti-VEGFα neutralizing antibodies. The next day, we determined the tumor sizes and repeated the injections, and the tumor sizes were measured again the following day. We observed that the supernatant from dNK cells derived from URSA donors exhibited a weaker promotive effect on tumor growth than that from dNK cells from NP or AK-RSA donors. T-EV treatment enhanced the growth-promoting effects of the supernatant from dNK cells from URSA donors, and this effect was blocked by anti-VEGFα neutralizing antibodies (Figure 5B-D). These data suggest that T-EVs enhance angiogenesis and trophoblast cell growth by promoting the secretion of VEGFα by dNK cells.

Fu et al. reported that dNK cells suppress TH17-mediated local inflammation via secreting IFN-γ at the maternal-fetal interface 11. Therefore, we test the effect of IFN-γ neutralization on Th17 induction after T-EV treatment (Figure 5E). Supernatants obtained from dNK cells from URSA donors and dNK cells from URSA donors incubated with T-EVs in the presence or absence of anti- IFN-γ neutralizing antibodies were incubated with CD4^+^T cells isolated from peripheral blood. The supernatants derived from URSA donors incubated with T-EVs inhibited Th17 induction and this effect was partially blocked by anti- IFN-γ neutralizing antibodies (Figure 5E, F). These data suggest that T-EVs inhibited Th17 induction by promoting the secretion of IFN-γ by dNK cells.

### T-EVs promote secretion of IFN-γ and VEGFα by NK cells in the uterus to maintain pregnancy via Qa-1 *in vivo*

**Figure 6:**
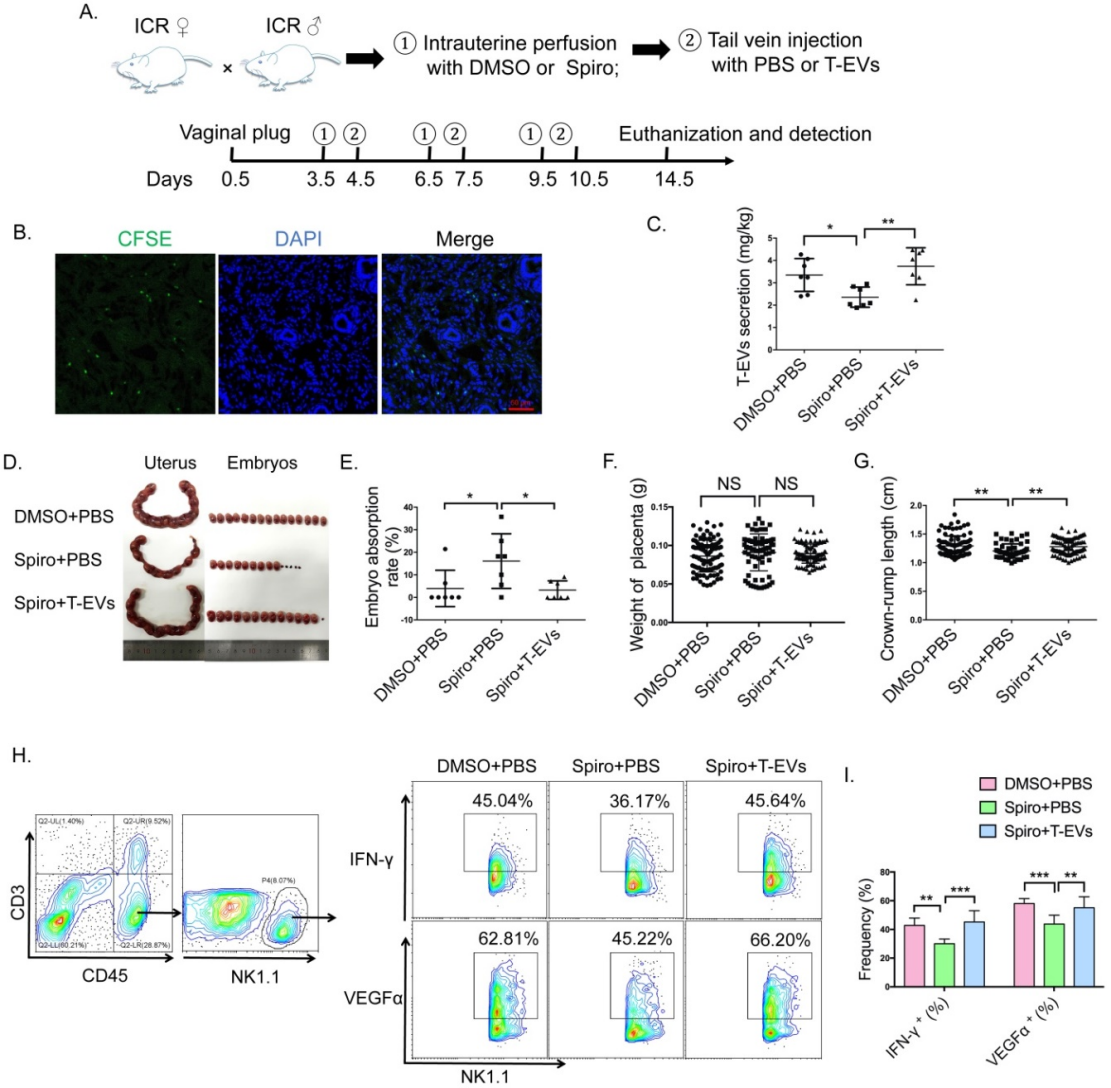
** The inhibition of T-EV secretion increased susceptibility to abortion. A.** ICR♀ × ICR ♂ mating combinations were established, and the detection of a vaginal plug was chosen to identify day 0.5 of gestation. ICR females received three intrauterine perfusions with DMSO or 50 µM spiroepoxide on days 3.5, 6.5 and 9.5 and three tail vein injections with PBS or 100 μg T-EVs on days 4.5, 7.5 and 10.5. The mice were euthanized on day 14.5. **B.** ICR female mice were injected with CFSE-labeled T-EVs via the tail vein. Twenty-four hours later, the mice were euthanized, and the uteri were dissected for immunofluorescence. Scale bar, 50 µm.** C.** T-EVs obtained from the different mouse models described above were quantified and analyzed.** D.** Representative photographs of the uteri and fetuses in the groups of mice described in **A. E.** The percentage of resorbed embryos was calculated as follows: resorbed embryos/total embryos x 100.** F.** The weights of the placentas from three groups of mice.** G.** The crown-rump length of the fetal mice from the three groups of mice. **H.** Representative contour images showing analysis of CD45, CD3 and NK1.1 expressions in uterus cells; intracellular expression of IFN-γ and VEGFα in CD45^+^CD3^-^NK1.1^+^ NK cells in uteri obtained from different groups of mice was detected by FCM and** (I)** analyzed statistically. Mean ± SEM (n=7, *P < 0.05, ** P < 0.01, *** P < 0.001; NS, not significant). Significance in (**F, G**) was determined by one-way ANOVA, with a Kruskal-Wallis posttest for multiple comparisons between DMSO + PBS (n=96), Spiro + PBS (n=73) and Spiro + T-EVs (n=96). The bar plot overlays in (**F, G**) depict the mean and SD.

Because T-EVs are involved in maintaining pregnancy, the inhibition of the endogenous production of T-EVs probably made the mice more susceptible to abortion. We confirmed the inhibitory effect of spiroepoxide on EV release from villous explants, and we found no obvious differences in the growth of villous explants without and with spiroepoxide treatment. Then, we applied spiroepoxide to study the effect of T-EVs on pregnancy *in vivo* (Figure 6A). To assess whether mouse T-EVs can traffic to the endometrium via peripheral blood, we intravenously transferred CFSE-labeled mouse T-EVs into ICR female mice and evaluated the distribution of these T-EVs in the uterus *in vivo*. Extensive distribution of the exogenic mouse T-EVs in the endometrium was observed (Figure 6B). After treatment with spiroepoxide, no mice died, their mental state did not change, and the quantity of T-EVs significantly decreased, confirming the inhibitory effect on the release of T-EVs *in vivo* (Figure 6C). As expected, the mice treated with spiroepoxide had more embryo absorption than their control group (Figure 6D and E), the placental weights (Figure 6F) were not significantly different among the three groups of mice, crown-rump lengths of the fetal mice of the mice treated with spiroepoxide were significantly shorter (Figure 6G) and the intracellular expression of VEGFα and IFN-γ in NK cells in the uterus decreased significantly (Figure 6H and I). Furthermore, the exogenous transfer of T-EVs abolished the effect of spiroepoxide (Figure 6C, D, E, G, H, I). These results suggest that the inhibition of T-EV production *in vivo* increases the susceptibility of mice to embryo absorption.

**Figure 7:**
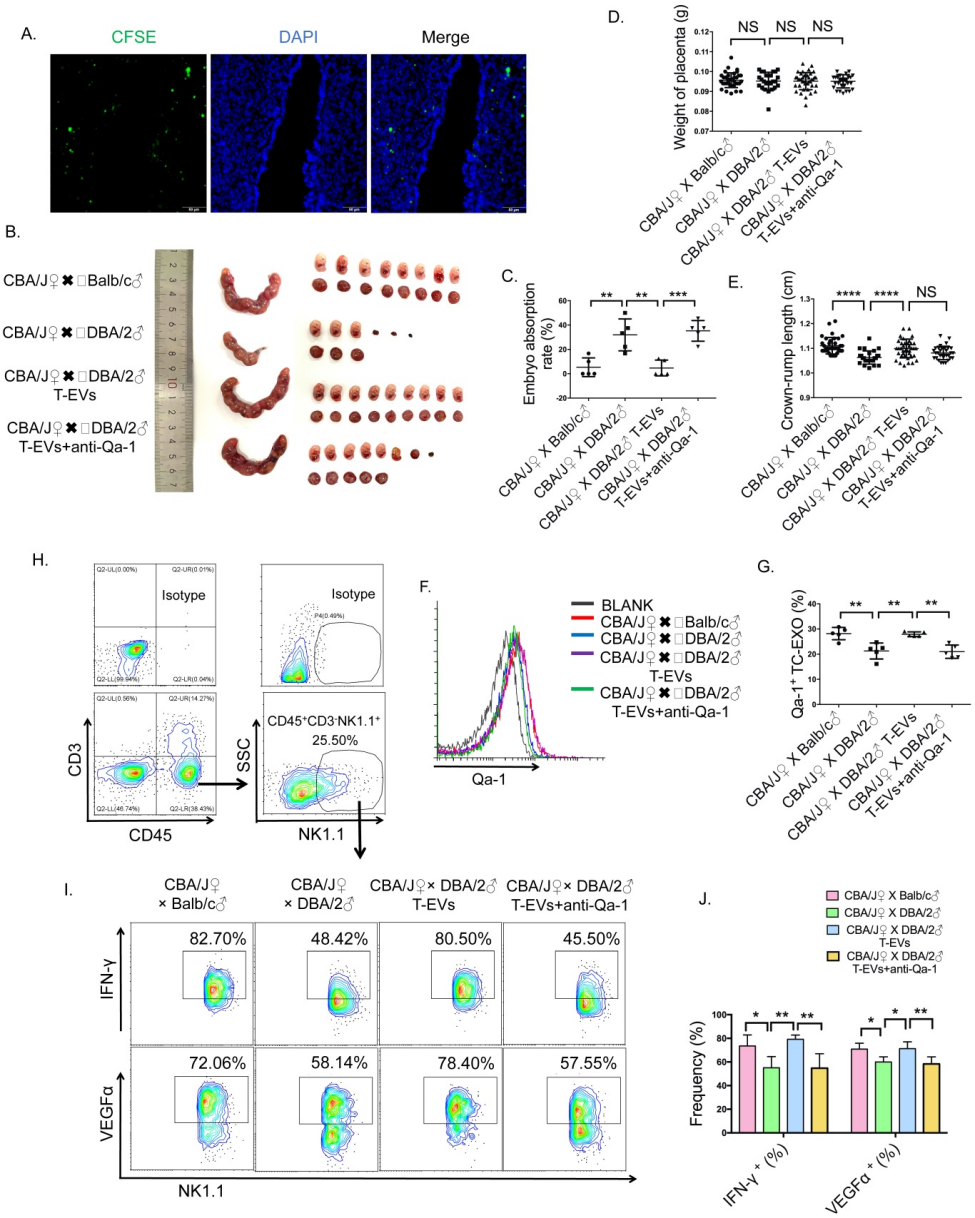
** T-EVs promoted the secretion of IFN-γ and VEGFα by dNK cells to maintain pregnancy via Qa-1 in an abortion-prone mouse model.** CBA/J ♀ × Balb/c ♂ mating combinations (normal pregnancy mouse model) and CBA/J ♀ × DBA/2 ♂ mating combinations (abortion-prone mouse model) were established, and the detection of a vaginal plug was chosen to identify day 0.5 of gestation. CBA/J females mated with DBA/2 males received three injections of PBS, 200 µg/mouse T-EVs or 200 µg/mouse T-EVs pretreated with an anti-Qa-1 Ab (100 µg/mL) on days 1.5, 3.5 and 5.5 via tail vein injection. **A.** CBA/J female mice were injected with CFSE-labeled T-EVs via the tail vein. Twenty-four hours later, the mice were euthanized, and the uteri were dissected for immunofluorescence. Scale bar, 50 µm. **B.** Representative photographs of the uteri and fetuses in the groups of mice described above. The percentage of resorbed embryos was calculated as follows: resorbed embryos/total embryos x 100. **C.** The percentage of embryo resorption in different mouse models was analyzed statistically. **D.** The weights of placentas from the three groups of mice. **E.** The crown-rump lengths of the fetal mice from the three groups of mice. **F.** T-EVs obtained from the different mouse models described above were combined with 4-µm latex particles to detect the level of Qa-1 using FCM (gray: isotype control; red: T-EVs from a control group mouse; blue: T-EVs from an abortion-prone model mouse; purple: T-EVs from an RSA mouse injected with T-EVs via the tail vein; green: T-EVs from an RSA mouse injected with T-EVs plus the anti-Qa-1 antibody via the tail vein). **G.** The percentage of Qa-1^+^ T-EVs was statistically analyzed (** *P* < 0.01, *** *P* < 0.001). **H.** Representative contour images showing analysis of CD45, CD3 and NK1.1 expressions in uterus cells. **I.** Intracellular expression of IFN-γ and VEGFα in CD45^+^CD3^-^NK1.1^+^ dNK cells in uteri obtained from different groups of mice was detected by FCM and (**J**) analyzed statistically. Mean ± SEM (n=5, *P < 0.05, ** P < 0.01, *** P < 0.001; NS, not significant). Significance in (**D, E**) was determined by one-way ANOVA, with a Kruskal-Wallis posttest for multiple comparisons between CBA/J ♀ × Balb/c ♂ (n=36), CBA/J ♀ × DBA/2 ♂ (n=26), CBA/J ♀ × DBA/2 ♂ T-EVs (n=40) and CBA/J ♀ × DBA/2 ♂ T-EVs+anti-Qa-1 (n=31). The bar plot overlays in (**D, E**) depict the mean and SD.

To confirm the function of T-EVs in regulating dNK cells and maintaining pregnancy *in vivo*, we used CBA/J female mice mated with DBA/2 male mice to establish abortion-prone mouse models, and CBA/J female mice mated with Balb/c male mice were used as controls. The embryo resorption rate of the abortion-prone model was 12.5-66.7%, and that of the control group was 0-16.67% (Figure 7B and C). The weights of the placentas (Figure 7D) were not significantly different among the three groups of mice. The crown-rump lengths of the fetal mice were significantly shorter in the abortion-prone model (Figure 7E). In addition, we also found that the Qa-1 level in T-EVs (Figure 7F and G) and the VEGFα and IFN-γ levels (Figure 7H and I) in dNK cells were significantly decreased in the abortion-prone model, which was similar to the phenomena observed in URSA patients.

To assess whether mouse T-EVs can traffic to the endometrium via peripheral blood, we intravenously transferred CFSE-labeled mouse T-EVs into CBA/J female mice and evaluated the distribution of these T-EVs in the uterus *in vivo*. Extensive distribution of the exogenic mouse T-EVs in the endometrium was observed (Figure 7A).

We intravenously transferred mouse T-EVs from CBA/J female mice in the control group to CBA/J female mice in the abortion-prone group and found that the exogenic mouse T-EVs ameliorated the embryo resorption rate and the crown-rump lengths of the abortion-prone model (Figure 7B, C and E), elevated the Qa-1 level in T-EVs extracted from the abortion-prone CBA/J female mice (Figure 7F and G), and facilitated VEGFα and IFN-γ (Figure 7H, I and J) expression in dNK cells. To determine the role of Qa-1 in the T-EV-mediated decrease in the embryo resorption rate and increases in the VEGFα and IFN-γ levels in dNK cells, T-EVs were incubated with an anti-Qa-1 blocking antibody before transfer. The protective effect of these T-EVs in limiting embryo resorption was somewhat decreased (Figure 7B and C), the elevation in the Qa-1 level induced by T-EVs was lost (Figure 7F and G), and the increases in the VEGFα and IFN-γ (Figure 7H, I and J) levels in dNK cells were reduced.

Together, these results suggest that T-EVs promote the secretion of IFN-γ and VEGFα by dNK cells to maintain pregnancy via Qa-1 *in vivo*.

## Discussion

During early pregnancy, the number of CD56^+^ dNK cells increases rapidly in the decidua, with these cells exhibiting low cytotoxicity compared to peripheral NK (pNK) cells, accompanied by the secretion of cytokines with multiple functions 48. Emerging evidence indicates an association between abnormalities in dNK cells and the occurrence of URSA 49. Fu et al. 9 identified uterine CD49a^+^Eomes^+^ NK cells that secrete growth-promoting factors (GPFs), which enhance fetal growth during critical early stages of fetal development. The GPF-secreting function of this NK cell subset is dependent on the crosstalk between HLA-G and ILT2 9. Gamliel et al. 10 reported an NK cell population from repeated pregnancies that expressed the receptors NKG2C and ILT2 and interacted with HLA-E and HLA-G, respectively, secreting IFN-γ and VEGFα to maintain pregnancy. However, they employed the MHC class I negative cell line 721.221, which was transfected to express HLA-E or HLA-G to engage the two receptors, and the result may be different from the real interaction between trophoblast cells and dNK cells under physiological conditions 10. To our knowledge, this study is the first to determine that HLA-E was secreted by trophoblasts via EVs to regulate the metabolism of human dNK cells in URSA patients. Our data suggest that HLA-E in T-EVs can facilitate glycolysis and OxPhos and thus the secretion of IFN-γ and VEGFα by dNK cells to maintain early pregnancy, which may be a useful supplement to previous research on the regulation of dNK secretion functions.

In previous reports, HLA-G was reported to play a dominant role in regulating the secretion functions of dNK cells, mainly through direct contact between trophoblasts and dNK cells 9,10. In this study, HLA-G was also secreted in response to T-EVs based on western blotting data (Figure 1C); however, the level of HLA-G was almost undetectable by FCM (Figure 1D), indicating that the HLA-G was located inside the EVs rather than on the surface. The effects of HLA-G cargo in T-EVs remain to be elucidated.

We found that HLA-E in trophoblast cells might interact with dNK cells via T-EVs since HLA-E was shown to be limited to the cytoplasm of trophoblast cells. Although surface expression of HLA-E on trophoblast cells has also been reported 50,51, another study found that HLA-E was located only intracellularly in villous trophoblast (VT) cells and only sporadically expressed in syncytiotrophoblast (ST) cells. In addition, HLA-E was detected in only some EVTs, and this expression also seemed to be confined to the cytoplasm 16. In this study, we found that the surface expression of HLA-E was very low; however, cytoplasmic staining of human villous trophoblast cells and the JEG-3 and HTR-8/Svneo cell lines indicated a substantial increase in HLA-E expression after treatment with IC fixation buffer. Immunofluorescence staining of villous tissue also revealed the greatest distribution of HLA-E in the cytoplasm. However, the molecular mechanisms by which HLA-E levels decrease in T-EVs from URSA patients need further study.

Villous tissue from early pregnancy mainly includes trophoblast cells, mesenchymal cells and a small amount of infiltrating immune cells 52. The EVs that we obtained from the villi were mainly derived from trophoblast cells, and we found no expression of immune cell markers, such as CD45, CD3, CD56, CD16 and CD11b, on EVs by FCM, suggesting that these EVs were not derived from immune cells. Western blotting indicated that the EVs did not contain Vimentin, which is a marker of mesenchymal cells; thus, the EVs were not derived from mesenchymal cells. The EVs contained large amounts of HLA-G, HLA-E and PLAP, suggesting that they were derived from villus trophoblasts.

King et al. 53 reported that the level of pNK cells in RSA patients is increased, but whether the number or percentage of dNK cells changes in URSA patients is still unclear. Lu et al. 54 reported that the percentage of CD3^-^CD56^bright^ dNK cells is decreased in RSA patients, while that of CD3^-^CD56^dim^ dNK cells is increased, but their sample number was very small, and they did not include AK-RSA patients as controls. A meta-analysis of studies showed no significant difference in the percentage of dNK cells between women with RSA and healthy controls 55, but this analysis also did not include AK-RSA patients. Our study not only indicates decreased levels of IFN-γ and VEGFα in dNK cells from URSA patients but also reveals that the levels of IFN-γ and VEGFα in dNK cells from AK-RSA patients did not change significantly compared to those from NP patients. In addition, there were positive correlations between the HLA-E level of T-EVs and the secretion of IFN-γ and VEGFα by dNK cells, suggesting that secretory dysfunction in dNK cells may participate in the occurrence and progression of URSA.

It is widely recognized that NK cells undergo dramatic metabolic reprogramming upon activation. Keating et al. 21 reported that OxPhos and glycolysis are required to support IFN-γ production in the CD56^bright^ subset of human peripheral blood NK cells. Keppel et al. 56 demonstrated that metabolism provides an essential second signal for the induction of IFN-γ production by activating murine NK cell receptors, whereas prolonged treatment with high-dose IL-15 eliminates the metabolic requirement for receptor stimulation. It was also revealed that mouse NK cells undergo upregulation of the rates of glucose uptake and glycolysis upon IFN-γ production and that mTORC1 activity is essential for attaining this elevated glycolytic state 23. However, which metabolic processes are required for IFN-γ and VEGFα secretion and how metabolic reprogramming occurs in human dNK cells during pregnancy are unclear. This study reveals that IFN-γ secretion by human dNK cells requires not only glycolysis, confirming previous studies, but also OxPhos, which was confirmed by the application of the ATP synthase inhibitor oligomycin. VEGFα production was also dependent on glycolysis and OxPhos in dNK cells. In addition, for the first time, we demonstrated that T-EVs promote IFN-γ and VEGFα secretion by dNK cells by regulating glycolysis and OxPhos, and mTORC1 participates in the process by which T-EVs promote glycolysis but not by which they promote OxPhos in dNK cells from URSA patients, providing new insights into dNK cell function during early pregnancy.

It is worth noting that glycolysis in dNK cells from not only URSA patients but also AK-RSA patients was significantly decreased, with that of the former being lower than that of the latter. It seems that the occurrence of AK-RSA is not only due to embryonic chromosomal abnormalities but may also be related to metabolic abnormalities in dNK cells. However, our data indicate that the secretion of IFN-γ and VEGFα by dNK cells from AK-RSA patients is not significantly different from that by dNK cells from healthy donors. Therefore, glycolytic abnormalities in dNK cells may be related to other functions of dNK cells, which have not yet been identified in this context.

dNK cells are an important source of multiple growth factors and cytokines, whose two main functions are promoting vascular remodeling and regulating trophoblast invasion 57. IFN-γ in the decidua, as a key regulator of uterine arterial remodeling secreted by dNK cells in early pregnancy, is critical for successful fetal development and pregnancy outcomes. dNK cells can promote immune tolerance by dampening inflammatory Th17 cells through the secretion of IFN-γ during early pregnancy, and this regulatory response is lost in RSA patients 11. Early work in mice demonstrated that IFN-γ plays a critical role in the initiation of uterine arterial remodeling, angiogenesis at implantation sites, and the maintenance of the decidua 58,59. dNK cells also produce large amounts of VEGFα, which is a proangiogenic growth factor that can favor angiogenesis and participate in uterine spiral artery remodeling in early pregnancy 60. Our data reveal that dNK cells facilitate angiogenesis and trophoblast cell growth by secreting VEGFα and inhibit Th17 induction by secreting IFN-γ, confirming previous studies. We may further study the effect of T-EVs on other cytokines secreted by dNK cells in the future.

The mating combination CBA/J×DBA/2 has been widely used to mimic RSA due to the characteristic high fetal resorption rate and normal karyotype 61, providing a useful system to investigate immune system-related mechanisms that lead to the rejection of the semiallogeneic embryo and to explore methods that prevent pregnancy failure 62. Many studies have suggested that fetal absorption in this model is due to an imbalance in Th1/Th2 cells, which can amplify inflammation and result in fetal rejection 63,64, while adoptive transfer of regulatory T cells (Tregs) can reestablish maternal immune tolerance and significantly reduce fetal resorption rates 65-67. However, dNK cell functions and T-EVs in the RSA model have not yet been well characterized. In our study, HLA-E levels in T-EVs and IFN-γ and VEGFα secretion by dNK cells in the RSA mouse model were significantly decreased, which was in accordance with the results for URSA clinical specimens; therefore, this model was appropriate for use in our study. Our data confirm that T-EVs promote IFN-γ and VEGFα secretion by dNK cells to maintain pregnancy via Qa-1 *in vivo*. In addition, our data suggest that IFN-γ and VEGFα secretion by dNK cells may be affected by immunological rejection.

The number of AK-RSA and URSA patients included in this study was small, which is a deficiency of this study. In addition, the effect of T-EVs on the other growth-promoting factors secreted by dNK cells was not included in this study. Therefore, we will continue to collect more specimens to study the pathological mechanisms of URSA in greater depth.

In summary, our data suggest that T-EVs containing HLA-E promote IFN-γ and VEGFα secretion by dNK cells by facilitating glycolysis and OxPhos via HLA-E and that the increase in glycolysis but not OxPhos in dNK cells induced by T-EVs is dependent on mTORC1 activation. This study elevates our understanding of how fetal crosstalk with the maternal immune system maintains fetal growth and demonstrates the potential of T-EVs as biologic agents for the treatment of URSA.

## Supplementary Material

Supplementary figures and tables.
