# Supplementary material for: Extracellular Vesicle-Mediated Secretion of HLA-E by Trophoblasts Maintains Pregnancy by Regulating the Metabolism of Decidual NK Cells
Source: Int J Biol Sci. 2021 Oct 22;17(15):4377–95. doi: 10.7150/ijbs.63390 (PMC8579460; doi:10.7150/ijbs.63390)
Supplement: Supplementary file 1 — Supplementary figures and tables. [file ijbsv17p4377s1.pdf]

Supplementary material

Table S1. Details of the NP, AK-RSA and URSA patients (** *P* < 0.01, *** *P* < 0.001, **** *P* < 0.0001, NS, not significant).

Parameters	NP	AK-RSA	URSA	*P* Value		
Maternal age (y)	31.04 ± 0.8220, n=24	32.19 ± 0.5494, n=16	30.35 ± 0.9812, n=17	NP vs URSA *P* =0.05933	AK-RSA vs URSA P=0.1189	NP vs AK-RSA *P* =0.3063
Gestation age (weeks)	6.58 ± 0.2030, n=24	6.73 ± 0.4179, n=16	6.60 ± 0.3748, n=17	NP vs URSA *P* =0.9615	AK-RSA vs URSA P =0.8104	NP vs AK-RSA *P* =0.7152
Number of miscarriage	0.0 ± 0.0, n=24	1.44 ± 0.1281, n=16	2.65 ± 0.2090, n=17	NP vs URSA *P* < 0.0001	AK-RSA vs URSA P < 0.0001	NP vs AK-RSA *P* < 0.0001
Number of live births	1.33 ± 0.0982, n=24	0.06 ± 0.0625, n=16	0.35 ± 0.2090, n=17	NP vs URSA *P* < 0.0001	AK-RSA vs URSA P =0.2043	NP vs AK-RSA *P* < 0.0001
Number of pregnancies	3.04 ± 0.2039, n=24	1.62 ± 0.1797, n=16	3.056 ± 0.2775, n=17	NP vs URSA *P* =0.9596	AK-RSA vs URSA P < 0.0001	NP vs AK-RSA *P* < 0.0001

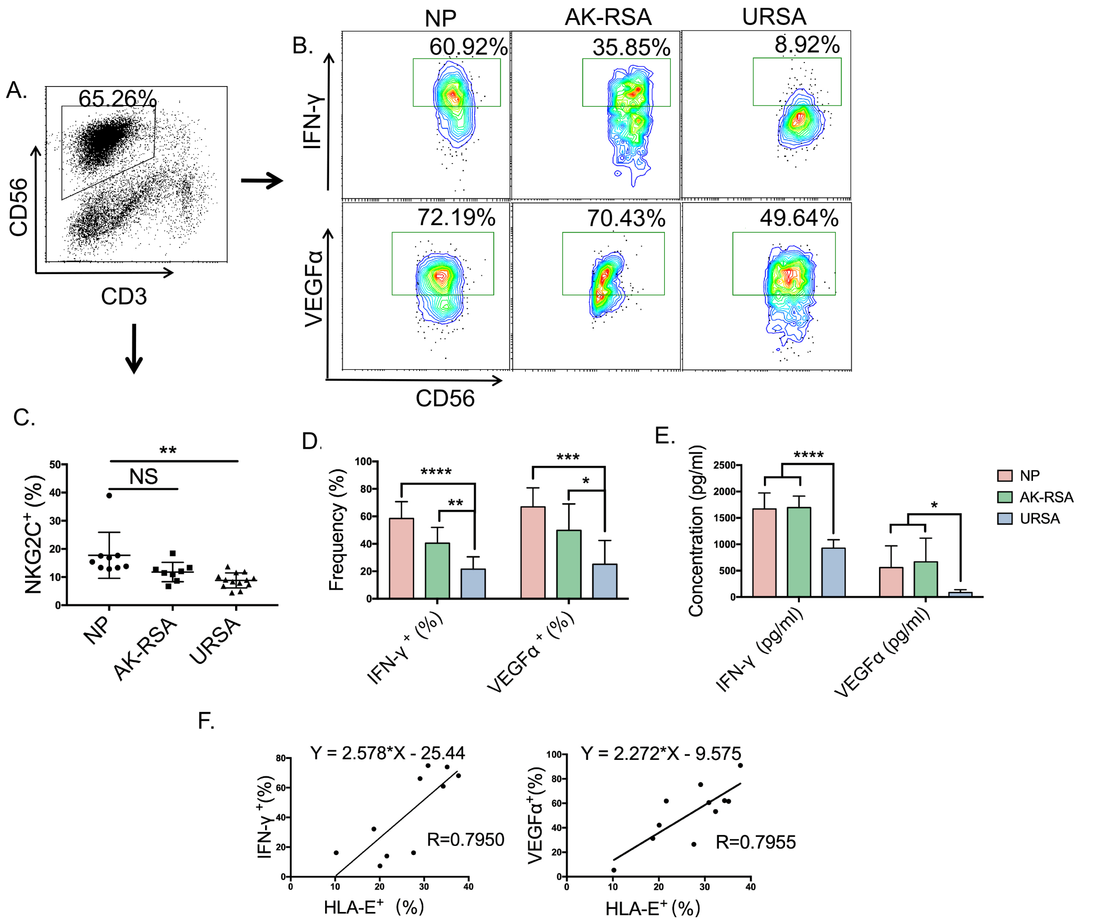

Figure S1. The secretion of IFN-γ and VEGFα by dNK cells from URSA donors decreased and was positively correlated with the levels of HLA-E in T-EVs.

A. B. dNK cells were purified from decidual tissues from NP, AK-RSA or URSA patients and seeded into 96-well plates at 1x10^5 cells/well in complete medium containing 50 ng/ml IL-15 for 48 h. Intracellular expression of IFN-γ and VEGFα in CD3$^-$CD56$^+$ dNK cells obtained from NP, AK-RSA or RSA patients was detected by FCM. **C.** The expression of NKG2C in CD3$^-$CD56$^+$ dNK cells obtained from NP, AK-RSA or RSA patients was detected by FCM and statistically analyzed. **D.** Intracellular expression of IFN-γ and VEGFα in

$CD3^{-}CD56^{+}$ dNK cells obtained from NP, AK-RSA or RSA patients was statistically analyzed. **E.** Supernatants of the dNK cells in **B.** were evaluated by ELISA, and the results were statistically analyzed. **F.** The correlations between the HLA-E level in T-EVs and intracellular IFN-γ and VEGFα expression in dNK cells were analyzed by Spearman correlation analysis using GraphPad Prism 6 (n=11). P values were generated by one-way analysis of variance (ANOVA) followed by the Newman-Keuls multiple comparison test using GraphPad Prism 6 (n=11 in NP group; n=11 in AK-RSA patient group; n=8 in URSA patient group, $^{*}P < 0.05$, $^{**}P < 0.01$, $^{***}P < 0.001$, $^{****}P < 0.0001$, NS, not significant).

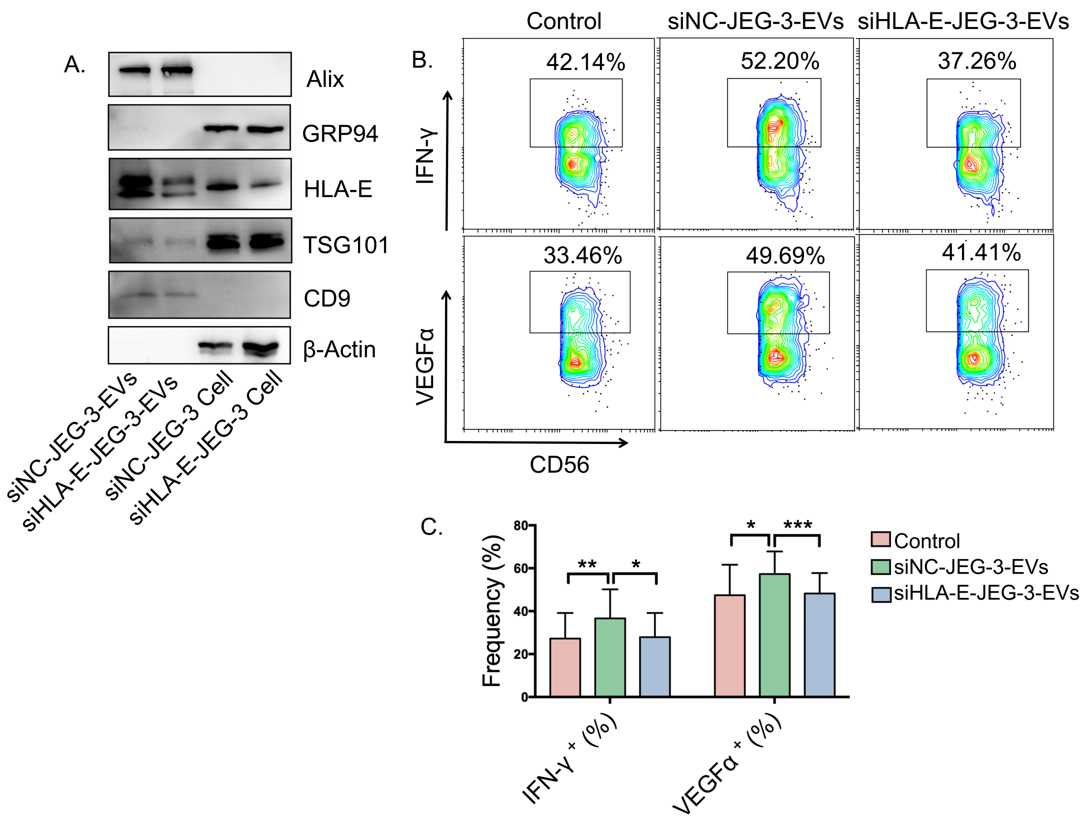

Figure S2. Exosomes from JEG-3 cells promote the secretion of IFN-γ

and VEGFα by dNK cells via HLA-E in vitro.

A. Western blot analysis of HLA-E, Alix, GRP94, TSG101 and CD9 in exosomes and cell lysates derived from negative control siRNA-treated JEG-3 cells (siNC-JEG-3) and HLA-E-specific siRNA-treated JEG-3 cells (siHLA-E-JEG-3). **B.** The intracellular expression of IFN-γ and VEGFα in dNK cells from URSA patients was detected by FCM and **C.** statistically analyzed. P values were generated by one-way analysis of variance (ANOVA) followed by the Newman-Keuls multiple comparison test using GraphPad Prism 6 (n=7, **P* < 0.05; ***P* <0.01; *** *P* < 0.001; **** *P* < 0.0001; NS, not significant).

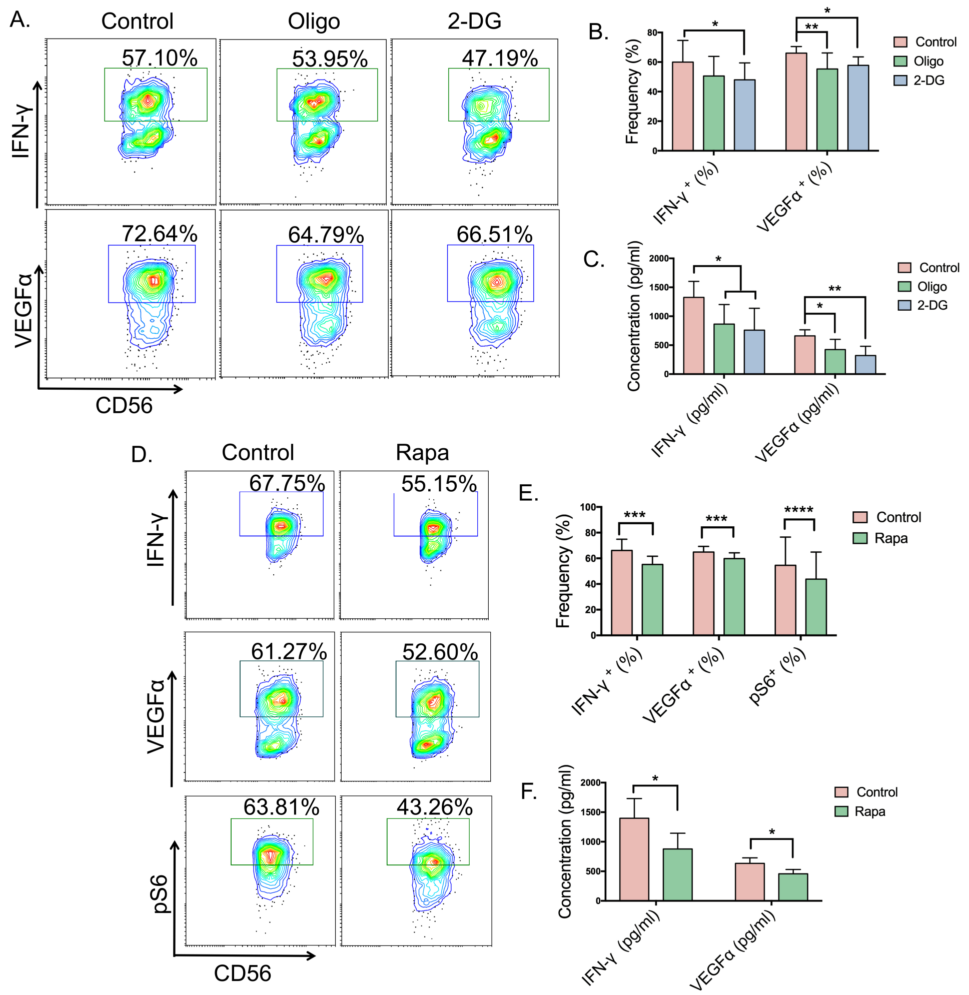

Figure S3. The secretion of IFN-γ and VEGFα by dNK cells from NP donors was dependent on cellular metabolism.

A. Intracellular expression of IFN-γ and VEGFα in dNK cells from NP donors treated with or without the glycolytic inhibitor 2-DG (1 mM) or ATP synthase inhibitor oligomycin (20 μM) was detected by FCM and statistically analyzed in **B. C.** Supernatants of the dNK cells in **a.** were detected by ELISA, and the results were statistically analyzed. **D.** Intracellular staining for pS6, IFN-γ and VEGFα in dNK cells treated with 10 nM mTORC1 inhibitor (rapamycin) was evaluated by FCM and statistically analyzed in **E. F.** Supernatants of the dNK cells in **D** were detected by ELISA, and the results were statistically analyzed. The data are representative of three independent experiments or are shown as the mean±s.e.m. pooled from three independent experiments. P values were generated by one-way analysis of variance (ANOVA) followed by the Newman-Keuls multiple comparison test using GraphPad Prism 6 (n=14, $*P < 0.05$; $** P < 0.01$; $*** P < 0.001$; $**** P < 0.0001$; NS, not significant).
